# When should global health actors prioritise more uncertain interventions?

**DOI:** 10.1016/S2214-109X(23)00055-4

**Published:** 2023-04

**Authors:** Leah Pierson, Stéphane Verguet

**Affiliations:** Harvard/MIT MD–PhD Program, Harvard Medical School, Harvard University, Boston, MA, USA; Department of Global Health and Population, Harvard T H Chan School of Public Health; Harvard University, Boston, MA, USA; Department of Global Health and Population, Harvard T H Chan School of Public Health, Harvard University, Boston, MA, USA

## Abstract

Global health actors use economic evaluations, including cost-effectiveness analyses, to estimate the effect of different interventions they might fund. However, producing reliable cost-effectiveness estimates is difficult, meaning organisations must often choose between funding interventions for which reliable predictions of efficacy exist and those for which they do not. In practice, many organisations appear to be risk-averse, favouring more certain interventions simply because they are more certain. We argue that this practice is not justifiable. Prioritising projects backed by greater evidence might often produce greater health benefits. However, a general tendency to prefer more certain interventions will cause global health actors to overlook opportunities to help less well-studied populations, support promising but complex interventions, address the upstream causes of illness, and conduct the most important impact evaluations. We argue that global health actors should instead adopt nuanced attitudes towards uncertainty and be willing to fund highly uncertain interventions in some cases. We further describe the considerations they should take into account in rendering these judgements.

## Introduction

Global health actors can use economic evaluations to set priorities.^1^ Specifically, organisations often rely on cost-effectiveness analysis, a method of predicting how much disease burden can be prevented at a given cost, to evaluate the potential effect of different interventions and prioritise more cost-effective ones.^2^ However, producing reliable cost-effectiveness estimates is difficult, meaning organisations must often choose between funding interventions for which reliable estimates of cost and effectiveness exist and those for which they do not.^3^ The less studied, more long term, or more complex an intervention is, the harder it is to reliably predict how well it will work.

In practice, many global health actors favour more certain interventions even when cost-effectiveness estimates fall within a similar range.^4^ An intervention is more uncertain when decision makers can less reliably predict the mean (ie, the expected) costs and effects of providing that intervention to a particular population. In cost-effectiveness analysis terms, our definition includes three types of uncertainty: first, parameter uncertainty, the uncertainty in estimating the parameter of interest (eg, the effect size of an intervention as measured in randomised controlled trials); second, heterogeneity, the variability between patients linked to attributes of those patients (eg, distinct population subgroups or socioeconomic groups); and third, structural uncertainty, the assumptions inherent to the decision model (eg, whether costs will increase linearly as an intervention is scaled).^5^

Global health actors’ tendency to prefer more certain interventions raises an important question: when is it justifiable for organisations to prioritise interventions that are known to be cost-effective instead of those that might be similarly cost-effective but for which cost-effectiveness estimates are less certain? In this Health Policy paper, we consider what attitudes these actors should adopt towards uncertainty, and when they should correspondingly prioritise more or less uncertain interventions.

We argue that global health actors can often maximise efficiency by prioritising more certain interventions, but that systematically doing so could cause them to forgo health gains and compound health inequities. Because of the potential downsides of unilaterally favouring more certain interventions, global health actors should adopt nuanced attitudes towards uncertainty, prioritising more certain interventions in some circumstances and less certain ones in others. We describe the considerations these actors should take into account in rendering these judgements.

## Background

Organisations such as The World Bank and the Bill & Melinda Gates Foundation have directed substantial funding towards global health projects.^6,7^ In an attempt to have as much impact as possible with limited funds, global health actors—organisations that work transnationally with the primary goal of improving health—have also invested heavily in measuring disease burden and estimating the potential impact of different health interventions.^8^ Using these data, some organisations generate cost-effectiveness estimates for different types of interventions. Such estimates are relevant to prioritisation decisions, although many organisations do not yet use them for prospective decision making. Other approaches (eg, benefit–cost analysis) are also used to set priorities. The issues we highlight here would apply to most other economic evaluation approaches, but we focus on cost-effectiveness analysis because it is more commonly used.

Global health actors using cost-effectiveness estimates preferentially fund interventions that are found to be more cost-effective. However, some cost-effectiveness estimates are more certain than others. For instance, estimating the cost-effectiveness of trachoma control measures might be relatively straightforward: one would estimate the prevalence of trachoma, the rate at which trachoma causes blindness, how well interventions preventing trachoma-induced blindness work, and the costs of these interventions. Cost-effectiveness analyses show that some interventions are orders of magnitude more cost-effective than others: if an organisation wanted to prevent disability associated with blindness, it could do so more efficiently by treating trachoma than by training guide dogs, as hundreds of cases of trachoma might be treated for the cost of training one guide dog.^9^ Cost-effectiveness analyses have thus allowed global health actors to prioritise interventions that are highly cost-effective, potentially saving millions of lives and improving many more.^10^ However, reliably predicting the cost-effectiveness of many interventions remains challenging, meaning some cost-effectiveness estimates are far more uncertain than others. There are several reasons for these differences (table).

First, cost-effectiveness estimates for more complex interventions (eg, interventions targeting structural causes of disease) are more uncertain. Although limited access to health care in many low-income and middle-income countries (LMICs) undermines health, evaluating interventions that target the social determinants of health or strengthen health systems can be challenging.^11^ For instance, assessing the cost-effectiveness of programmes aimed at increasing the number of physicians in LMICs requires estimating many parameters, each of which can be challenging to infer. Large numbers of physicians trained in LMICs emigrate elsewhere, but their reasons for emigrating vary, rendering predicting the effects of a given intervention on emigration rates difficult.^12^ Additionally, quantifying the effects of physician emigration on population health and evaluating how policies (eg, raising physicians’ salaries) affect migration has proven challenging. Although such evaluations are crucial, they are data-intensive to conduct. Consequently, cost-effectiveness analyses of more complex interventions tends to be less certain.^13^

Second, some interventions are less generalisable than others, leading to less reliable cost-effectiveness estimates when implementing or scaling up programmes in new settings. Although differences across populations and settings will increase the uncertainty of all interventions being implemented in new contexts, how well some interventions will translate to new settings is easier to predict than others. A global health actor planning to distribute long-lasting insecticidal nets in a new community could adjust the cost-effectiveness estimates by comparing differences in malaria incidence and distribution costs between the original and new setting. Yet, such simple adjustments are more difficult for other interventions. Sex education might work well in one context, but not in a context in which it is viewed as culturally inappropriate, and adjusting estimates based on local attitudes towards sex education is hard. Thus, evidence for some interventions will support implementing them in new settings, whereas evidence for others will be highly context-specific.

Third, assessing the cost-effectiveness of less well-studied interventions is difficult. A cost-effectiveness estimate is only as reliable as the data upon which it is based, but data might be absent or unreliable because an intervention is novel, or because limited research on that intervention has been conducted. Askell^14^ illustrates this point by considering the decision of whether to fund a novel intervention to reduce the prevalence of mosquitoes that transmit malaria. The novel intervention could outperform well-studied interventions (eg, long-lasting insecticidal nets), or prove ineffective. In Askell’s example, both the novel intervention and long-lasting insecticidal nets have the same point estimate of cost-effectiveness (around US$3000 per life saved). However, the novel intervention has a wider confidence interval: it could be far more cost-effective, or not work at all. In general, the less studied an intervention is, the less reliable estimates of its cost-effectiveness will be.

Finally, reliably estimating the cost-effectiveness of more future-oriented interventions is hard. For instance, cost-effectiveness estimates of pandemic preparedness interventions depend on unknown features of future pandemics, such as a pathogen’s transmissibility and virulence.^15^ Even during a pandemic, estimating these parameters is challenging, and predictions about future pandemics are necessarily more speculative. Thus, cost-effectiveness estimates will be more uncertain for interventions that would be used further in the future or that target more unpredictable health threats.

Many approaches have been developed to quantify the uncertainty of cost-effectiveness estimates. Most commonly, global health actors rely on sensitivity analyses to determine how robust an estimate is to alternate input values and parameters.^5^ For instance, in assessing the cost-effectiveness of an antiretroviral treatment programme for patients with HIV, an organisation might wish to know its effect with different medication adherence rates. Sensitivity analyses would allow that organisation to assess the likely cost-effectiveness of the programme if patients took their medications 10, 50, or 90% of the time. If the estimated cost-effectiveness shifted substantially based on alternate plausible adherence rates, the programme would be more sensitive, and hence less certain. If an organisation happened to know that adherence rates for a given population tended to be between 40 and 60%, this rate would make the cost-effectiveness model less sensitive, leading to more reliable estimates. When taking on cost-effectiveness analyses, sensitivity analyses are often conducted; however, how global health actors ought to consider this information is not yet clear.

## Ethical objectives in global health priority setting

Crucially, a global health actor’s ability to quantify uncertainty does not tell an actor what to do with this information, just as calculating a cost-effectiveness estimate does not answer the question of why cost-effectiveness matters, or when organisations ought to deviate from simply funding the most cost-effective interventions. Determining what global health actors’ attitudes towards uncertainty should be requires first clarifying the ethical objectives that should guide priority setting; when prioritising more certain interventions helps achieve these objectives, organisations should prioritise them; when it does not, they should not.

Two fundamental principles should drive global health actors’ decisions: they should prioritise interventions to the extent that those interventions are efficient and to the extent that they are equitable. Specifically, organisations should aim to maximise expected health gains and benefit more disadvantaged groups, including those who are sicker or more marginalised. Because these principles are widely accepted as important for priority setting, we will not defend them at length. We will also table discussion of the appropriate trade-offs between them. Although other ethical considerations—such as reciprocity and solidarity—might at times be relevant, we focus on efficiency and equity due to their strong links with uncertainty.^16-18^

Several points are worth clarifying. We do not rely on any specific conception of disadvantage or what maximising health benefits means. For instance, some global health actors might choose to maximise lives saved whereas others might choose to minimise disability-adjusted life-years. Because cost-effectiveness analysis is often used to quantify only an intervention’s direct costs and health effects, for simplicity (without the loss of generalisability), we focus here on the health benefits, rather than the broader social and economic benefits, these interventions yield. A global health actor prioritising the most cost-effective interventions would be aiming to maximise efficiency. Although some other economic evaluation tools account for distributional effects, they are not as widely used.^19^ As a result, global health actors who use cost-effectiveness analysis but want to reduce inequities would separately consider the disadvantage of potential beneficiaries.

Although global health actors should prioritise more efficient and equitable interventions, they are subject to several constraints. First, they should respond to the preferences of the populations they aim to support. Some populations might be more tolerant of uncertainty in general or in certain circumstances (eg, because they trust a given organisation). When possible, organisations should assess and respect local attitudes towards uncertainty.

Second, outside organisations should minimise interference with local decision making structures, as legitimate government ministries or other stakeholders might be better positioned to make particular decisions.

Finally, global health actors should generally adhere to their mandates. The Malaria Consortium’s mission is “to save lives and improve health in Africa and Asia, through evidence-based programmes that combat targeted diseases and promote universal health coverage”.^20^ This mission constrains the Malaria Consortium’s actions in that it requires the organisation to focus on populations in Africa and Asia. Even if the Malaria Consortium could identify a more efficient programme in South America, funding such a programme would be at odds with its mission.

If global health actors frequently find themselves unable to prioritise efficient and equitable projects because of their mandates, leading highly valuable projects to go unfunded, they should consider revising their mandates. However, organisations cannot change their missions overnight, as doing so would require taking appropriate legal steps, informing donors, developing new partnerships, and so on. Thus, we treat global health actors’ mandates as fixed, and assume organisations should adhere to them when making prioritisation decisions.

In the remainder of this paper, we consider what attitudes towards uncertainty would allow global health actors to more reliably identify efficient and equitable interventions.

## Uncertainty and efficiency

In many, but not all, cases, prioritising more certain interventions can produce greater health benefits than prioritising less certain interventions. Global health actors should thus adopt nuanced attitudes towards uncertainty depending on the specific features of a prioritisation decision (panel 1).

### Are sceptical prior beliefs warranted?

Holden Karnofsky—a Co-Founder of GiveWell, an organisation that searches for “charities that save or improve lives the most per dollar”—has advocated for prioritising more certain interventions, writing: “we generally prefer to give where we have strong evidence that donations can do a lot of good rather than where we have weak evidence that donations can do far more good”.^21^ This stance can be justified if one thinks cost-effectiveness analyses should not be taken at face value. When GiveWell undertook a detailed investigation of a cost-effectiveness analysis produced by the *Disease Control Priorities in Developing Countries* (second edition) it found that the cost-effectiveness of deworming was greatly over-estimated.^22^ Fact checking of this sort rarely occurs, but such inaccuracies should make us question the reliability of this type of analysis for interventions that are harder to assess and verify.

Organisations should thus gauge the expected health benefits of an intervention by combining two pieces of information: an estimate of its cost-effectiveness and an organisation’s prior belief about how well it will work. Because cost-effectiveness estimates for some interventions are less reliable than others, organisations need to weigh their prior beliefs more heavily when attempting to predict the true value of more uncertain interventions. Although cost-effectiveness analyses theoretically could incorporate Bayesian adjustments for prior beliefs, in practice, they rarely do.^21^

Karnofsky argues that global health actors should generally be sceptical about novel interventions, as most new interventions will not be among the most cost-effective. Interventions that appear highly cost-effective in one study but are backed by little data are likely to be less cost-effective in practice. If cost-effectiveness estimates are often inaccurate, and if global health actors ought to hold sceptical prior beliefs about the effectiveness of interventions, then prioritising more certain interventions when cost-effectiveness estimates are similar will tend to maximise health benefits.

Notably, Karnofsky’s argument for supporting interventions with more certain cost-effectiveness estimates hinges on an empirical claim: that sceptical prior beliefs are warranted. But this claim does not apply equally in all areas: in some arenas, uncertain interventions more reliably prove effective, and more optimistic prior beliefs might be indicated. For instance, new vaccines are ten times more likely than new cancer drugs to progress from phase 1 trials to approval.^23^ At a minimum, the consistently superior performance of some types of interventions or organisations means that differing degrees of scepticism are warranted, depending on the intervention being considered. For some disease areas or intervention types, cost-effectiveness estimates for uncertain interventions should probably not be tempered by sceptical prior beliefs. For global health actors to determine when scepticism is warranted can be challenging, yet they can attempt to do so by, for instance, considering how similar interventions within a given arena have done. Because global health actors often focus on specific diseases (eg, malaria) or intervention types (eg, immunisations), and repeatedly partner with the same organisations, they might be well positioned to make these assessments.

Assuming global health actors can identify these patterns, and can do so reliably, actors in areas where uncertain interventions have performed just as well as more certain ones have no reason to prioritise more certain interventions. Indeed, in these areas, organisations might forgo expected health benefits by favouring more certain interventions.

### Will valuable information be gained?

Askell acknowledges the merit of Karnofsky’s argument, writing “a skeptical prior about interventions will cause us to have a lower estimate of the effectiveness of an intervention that lacks evidential support”.^14^ However, putting aside this instrumental justification for supporting more certain interventions, Askell suggests that “all else being equal, we should expect to derive more value from investing in interventions for which we have less evidential support”.^14^

When the expected values of two interventions are comparable, Askell argues that there is no reason to prefer the more certain one, as across many interventions, the predicted value of the interventions will tend to approach the true value of those interventions. As a result, choosing interventions with the greatest expected value will, across time, maximise cost-effectiveness. When donors place prima facie value on certainty, they sacrifice health gains they might otherwise have reaped.

Askell further argues that if two interventions are expected to have similar value, we should favour the more uncertain one because “the information value we gain from investing in an intervention with less evidential support is generally greater because our estimates of the value of investing in these interventions are generally of higher variance…and of lower resilience”.^14^ In other words because we know less about uncertain interventions, we can potentially gain more valuable information from funding them. Askell therefore concludes that when interventions have comparable expected value, organisations should prioritise those with less evidential support.

Askell’s argument is theoretically compelling. Nonetheless, to produce formal expected value estimates, organisations would need to contend with the formidable task of reconciling cost-effectiveness estimates and prior beliefs. Instead, organisations typically do not produce expected value estimates, instead relying on cost-effectiveness estimates. And as discussed, cost-effectiveness estimates based on little data can often be biased upwards relative to an intervention’s true effects.

In practice, choosing the interventions with the most promising cost-effectiveness estimates (without accounting for prior beliefs) would be unlikely to maximise the value across all interventions; it would simply favour more uncertain ones, many of which might ultimately prove less cost-effective than known interventions. Moreover, even if a global health actor successfully reconciled cost-effectiveness estimates with prior beliefs, a policy of choosing interventions with the most promising expected value estimates would, in practice, be a policy of favouring more certain interventions. After all, if we accept that sceptical prior beliefs should be used, the cost-effectiveness estimate for an uncertain intervention must be better than that for a more certain intervention to produce a comparable expected value estimate.

Furthermore, although global health actors theoretically stand to learn more about interventions they know less about, in practice there could be a causal relationship between present uncertainty and the amount of information that would be gained from funding an intervention. For instance, if a cost-effectiveness estimate for an intervention is highly uncertain because it is extremely difficult to study or because its benefits would only be realised far in the future, global health actors might gain little information from funding it. In these cases, global health actors might learn less from funding uncertain interventions. Although gaining information that could guide future investments is valuable, and global health actors stand to learn more about interventions they presently know less about, whether or not funding more uncertain interventions would systematically lead to greater information gains in practice is not clear.

Notably, uncertainty is not static. When the major drivers of uncertainty in a cost-effectiveness estimate are known, organisations can conduct research to reduce these key uncertainties.^24^ When research is likely to lead to important new information about the cost-effectiveness of an intervention, global health actors should consider funding this research, as doing so could help them make more prudent investments in the long term, leading to greater health gains.

### Is a given kind of intervention inherently uncertain?

Some interventions are uncertain because they have not been studied sufficiently. When an intervention could be rigorously evaluated, but has not been, global health actors can reasonably deprioritise it until more data is collected. By contrast, global health actors should not deprioritise entire classes of interventions—including more complex, future-oriented, and less generalisable ones—because these interventions are inherently hard to assess, and correspondingly more uncertain. A bias towards certainty can lead funders to favour simple interventions that are backed by substantial data, and thus reliable cost-effectiveness estimates, instead of uncertain but potentially transformative ones. Although prioritising more certain interventions is often reasonable, organisations should not deprioritise more cost-effective interventions or overlook interventions for which cost-effectiveness estimates cannot reliably be generated simply because assessing their benefits is difficult. For instance, global health actors should not avoid funding advocacy work, public health education, or innovative research simply because estimating their cost-effectiveness is hard. Rather, cost-effectiveness analysis might not be the right tool for comparing the most uncertain classes of interventions. In these cases, global health actors might consider using less granular approaches to make comparisons, rather than deprioritise interventions for which cost-effectiveness estimates seem unreliable or do not exist. For instance, Open Philanthropy, which makes large grants to global health organisations each year, selects focus areas using the Importance-Neglectedness-Tractability framework.^25^ A problem is more important when it affects more people, it is worse for each person it affects, and addressing it could provide substantial benefits; a problem is more neglected when it receives little attention relative to its importance; and a problem is more tractable when there are “clear ways in which a funder could contribute to progress”.^26^ The framework can sometimes serve as a substitute for cost-effectiveness analysis when cost-effectiveness estimates are not available or would be very difficult to generate.^27^

### Will it be possible to change course?

When withdrawing funding for an intervention in light of new information would be easy, global health actors should tolerate greater uncertainty. For instance, an organisation providing antiretroviral treatment to patients with HIV cannot withdraw funding without putting beneficiaries at risk. In other cases, withdrawing support might be politically infeasible. Before supporting irreversible interventions, global health actors should be highly confident that they are cost-effective. Funding an intervention that turns out not to be cost-effective is a bigger mistake when this decision cannot be revised.

## Uncertainty and equity

Global health actors’ attitudes towards uncertainty can also mitigate or compound inequities, depending on the type of uncertainty involved (panel 1). Uncertainty due to limited data or a lack of research on specific populations (or inability to generalise research results to those populations) can be linked to inequities. Organisations can thus compound inequities by minimising these types of uncertainty.

Historically, most research funds have been directed towards studying diseases that predominantly affect more advantaged populations (ie, the 10–90 gap).^28^ As a result, far more is known about some diseases than others. For instance, in the USA, research funders devote ten times as much funding per patient to cystic fibrosis, a disease that primarily affects White patients, than to sickle cell disease, which disproportionately affects Black patients.^29^ Most likely because of this disparity, more articles on cystic fibrosis have been published and more treatments for it have been approved than for sickle cell disease. Insufficient funding into neglected diseases could cause global health actors to underestimate the scope of some problems or lack tools for mitigating them.

In addition, even when research is funded, disadvantaged populations often have poor access to clinical trials and other research opportunities (panel 2). For instance, people who are not White, on low-incomes, homeless, live in rural areas, or have disabilities are under-represented in research.^39,40^ This phenomenon can lead to unusually high uncertainty about the benefits promising interventions could provide to disadvantaged populations. A paucity of data on underserved populations means interventions aimed at improving their health might tend to be more uncertain. If research output is a major determinant of the reliability of cost-effectiveness estimates, and if global health actors tend to favour interventions for which there are more reliable cost-effectiveness estimates, organisations might inadvertently compound inequities in research funding by prioritising more certain interventions.^41^

Admittedly, uncertainty in cost-effectiveness estimates is often not linked to a dearth of funding for diseases affecting disadvantaged groups. For instance, challenges associated with estimating the cost-effectiveness of future-oriented interventions, such as pandemic preparedness measures, have less to do with research funding disparities and more to do with difficulties inherent to predicting the future. Given this fact, using uncertainty as a heuristic by which to identify interventions that will reduce inequities would be a mistake. Global health actors should instead strive to identify the likely beneficiaries of the programmes they plan to fund, and should prioritise those that will aid more disadvantaged groups. Interventions that benefit more disadvantaged populations might often be more uncertain, in which case global health actors should tolerate higher levels of uncertainty than they might otherwise.

Finally, the equity implications of minimising uncertainty due to complexity are unclear. On the one hand, disadvantaged groups have worse access to basic interventions, such as immunisations. Expanding such access could thus improve the health of worse-off populations. On the other hand, populations experience poor health because of broader injustices, including economic inequalities and discriminatory laws, which are often complex and entrenched. If global health actors opt not to fund interventions addressing the upstream causes of poor health because such interventions are uncertain, they might do less than they could to mitigate inequities. For global health actors not to address such injustices simply because they cannot reliably estimate, for instance, the cost-effectiveness of lobbying for higher cigarette taxes, would be a mistake.

## Conclusion

Global health actors should not prioritise more certain interventions simply because they are more certain, as doing so could cause them to overlook opportunities to aid less well-studied populations, address the structural causes of illness, conduct valuable impact evaluations, and identify promising new interventions. Instead, global health actors should strive to generate greater health benefits and support more disadvantaged populations, and these goals should determine their attitudes and actions towards uncertainty in each case. To this end, global health actors should tolerate more uncertainty when sceptical prior beliefs about an intervention are not warranted, when implementing an intervention will generate information that could guide future investments, when a given kind of intervention is inherently uncertain, and when a decision is revisable. In addition, global health actors striving to reduce inequities might often need to tolerate greater uncertainty, given the lack of research on many disadvantaged populations. Global health actors’ attitudes towards uncertainty should thus be informed by the specific features of a prioritisation decision and the nature of the uncertainty underlying the construction of a cost-effectiveness estimate.

## Figures and Tables

**Table:** Sources of uncertainty in the evaluation of global health interventions

	Example	Implications of minimising uncertainty
Complexity	Intervention to increase the number of doctors in low-income and middle-income countries	Complex interventions deprioritised
Generalisability	Sex education programme	Less generalisable interventions deprioritised; interventions in new settings deprioritised
Imperfect data	Experimental intervention to prevent malaria	Novel or less well-studied interventions deprioritised
Unpredictability	Pandemic preparedness intervention	Future-oriented interventions deprioritised

## Panel 1: Criteria for determining whether to prioritise more or less uncertain interventions

### Are sceptical prior beliefs about an intervention warranted?

If a partner organisation has a good track record of training community health workers for chronic diseases, and could additionally train them to combat mental illnesses, uncertainty about the efficacy of the latter intervention should be tempered by high confidence in the organisation responsible for implementing it.

### Will information be gained that will help guide future investments?

When deciding whether to prioritise health-care vouchers versus unconditional cash transfers, more research having been done on the latter provides an additional reason to prioritise the former, assuming research will be conducted that will resolve key uncertainties.

### Is a given kind of intervention inherently uncertain?

When deciding whether to prioritise lobbying for a policy requiring schools to serve healthy meals or instead distributing healthy meals to families, global health actors should not deprioritise the former intervention simply because it is hard to predict the effects of advocating for policy changes.

### Will minimising uncertainty compound inequities?

Low-resource settings often have weaker health information systems. Infrastructural challenges can cause estimates of disease burden to be more uncertain, making predicting the effects of health interventions difficult. Global health actors should not avoid working in settings with weak health information systems simply because the benefits of interventions in these settings might be more uncertain.

## Panel 2: Inequities in access to research

In the USA, far more clinical trials of novel pharmaceuticals are funded by private companies than by the federal government.^30^ Companies not only devote relatively few resources to researching drugs that could benefit low-income populations—who might be unable to afford the drugs these trials yield—but also towards recruiting underserved populations to the studies they do fund.^31,32^ One result of this situation is that participants in clinical trials of new drugs are overwhelmingly White, despite evidence that populations who are not White are often as interested in research but face greater barriers to participating.^33,34^ In trials of aducanumab, a drug for Alzheimer’s disease, less than 1% of participants identified as Black, while more than three quarters identified as White, despite the fact that older Black adults are more than twice as likely as older White adults to have Alzheimer’s disease.^35,36^ When populations are under-represented in research, it can lead to greater uncertainty about the benefits new treatments will provide to members of those populations. Because cost-effectiveness estimates of new drugs are based on trial data, these estimates will tend to be more reliable for the groups that were well represented in those trials.^37^ Recent research also suggests that physicians might be less willing to prescribe novel treatments to patients from groups who are under-represented in clinical trials.^38^
